# An Integrative Genetic Strategy for Identifying Causal Genes at Quantitative Trait Loci in Chickens

**DOI:** 10.3390/ani16020155

**Published:** 2026-01-06

**Authors:** Akira Ishikawa

**Affiliations:** Laboratory of Animal Genetics and Breeding, Graduate School of Bioagricultural Sciences, Nagoya University, Nagoya 464-8601, Japan; ishikawa@agr.nagoya-u.ac.jp; Tel.: +81-52-789-4101

**Keywords:** QTL, GWAS, integrative approach, gene prioritization, causal genes, chickens

## Abstract

Background: Identifying causal genes underlying quantitative trait loci (QTLs) remains challenging due to small effect sizes and the prevalence of non-coding variants. Although multi-omics integration frameworks such as eQTL- and epigenomic-based approaches and TWAS have advanced gene prioritization, their application in poultry and livestock is often constrained by limited reference panels and tissue resources. This review introduces a cost-effective F_2_-based integrative framework and compares it with existing multi-omics strategies. Methods: The proposed framework combines QTL remapping, transcriptome analysis, haplotype frequency comparison, association analysis, and conditional correlation analysis within a single workflow. Causal analysis and quantitative complementation tests using knockout birds are incorporated to identify causal genes. Results: By reusing the original F_2_ population employed for QTL mapping, this approach enables hypothesis-independent gene prioritization without requiring additional fine-mapping crosses. Its effectiveness is demonstrated through comparison with conventional multi-omics methods, and the integration of causal analysis and quantitative complementation testing provides robust genetic evidence for pinpointing causal genes. Conclusions: This F_2_-based framework efficiently prioritizes and verifies causal gene candidates directly within the mapping population, offering a cost-effective alternative to multi-omics approaches that require large-scale resources. It is broadly applicable to diverse chicken crosses and readily transferable to other small livestock species and model organisms.

## Simple Summary

Quantitative trait locus (QTL) analysis and genome-wide association studies (GWASs) have revealed many genomic regions associated with important quantitative traits in chickens. However, identifying causal genes within these regions is still difficult. Recently, we established a new integrative genetic strategy that combines multiple analyses to efficiently narrow down candidate genes. By adding causal analysis and quantitative complementation testing, this approach can identify the causal genes underlying quantitative traits. This review introduces the concept of this strategy and its advantages over conventional gene prioritization approaches.

## 1. Introduction

In animals, including poultry, livestock, and humans, most genetically determined phenotypic traits of agricultural, medical, and biological importance are quantitative in nature. These traits are influenced by multiple genetic loci, termed QTLs, as well as environmental factors and QTL-by-environment interactions. As reviewed by Miles et al. [1], two common approaches are used to map QTLs to chromosomal regions, both relying on statistical associations between genotypes at genetic marker loci and phenotypic values. One is GWASs, which are typically applied in outbred populations such as large livestock and humans. The other is genome-wide QTL analysis, often referred to simply as QTL analysis, which is performed in model animals and small livestock such as chickens and pigs, using segregating populations derived from three-generation pedigrees or designed crosses.

These two QTL-mapping approaches have identified numerous QTLs associated with quantitative traits across almost all animal chromosomes. However, pinpointing the causal genes and underlying genetic variants within these QTL regions remains a major challenge for several reasons. Most QTLs exhibit relatively small phenotypic effects, encompass broad regions with wide confidence intervals and substantial linkage disequilibrium (LD), and are predominantly located in non-coding genomic regions [2,3,4,5,6,7,8]. Identifying the causal genes and variants underlying these QTLs is critical for unraveling the complex genetic basis of quantitative traits, particularly those of economically important traits in poultry and livestock. These findings can be directly applied to tailored genetic improvement programs in these species.

Various single analytical methods have been developed for candidate QTL gene prioritization, based on positional cloning, gene expression profiling, and functional annotation. However, as highlighted in previous reviews [2,5], these single methods often struggle to reduce the number of candidates to only a few plausible genes out of the hundreds located within a given QTL region. To address this limitation, integrative approaches that combine multiple omics data, such as expression QTL (eQTL) mapping, epigenomic data, and comparative genomics, have been developed and applied to candidate gene selection in humans [8,9,10], livestock [11], and chickens [7]. In this review, I provide a systematic comparison between our proposed framework and these established multi-omics integration strategies, emphasizing similarities in analytical concepts, differences in data requirements, and situations where each approach is most effective. This perspective is essential because, although current multi-omics frameworks are highly powerful, they often require large sample sizes, extensive tissue collections, or population-scale resources that are not always available in poultry and livestock species.

Recently, we established a new integrative approach in chickens that combines QTL remapping, RNA-sequencing (RNA-seq) analysis, reverse transcription quantitative PCR (RT-qPCR) validation, haplotype frequency comparison, association analysis, and conditional correlation analysis [12]. This method is hypothesis-free and cost-effective, utilizing a segregating F_2_ population derived from a cross between two chicken breeds with different phenotypes, thereby eliminating the need for additional QTL fine-mapping. Using this strategy, we successfully prioritized only two candidate genes from 333 genes located within the confidence interval of a QTL on chromosome 4 associated with innate fear behavior. This was achieved by analyzing two extreme groups exhibiting the highest and lowest behavioral values, derived from an F_2_ population generated between the native Japanese Nagoya (NAG) breed with a timid temperament and the White Leghorn (WL) breed with a normal control temperament.

This review presents the conceptual framework of our new prioritization strategy and describes its integration with causal analysis and quantitative complementation tests, as previously employed in our mouse study [13]. Together, this integrative approach enables the identification of causal genes underlying quantitative traits. Additionally, by systematically contrasting this workflow with existing multi-omics integration frameworks, I aim to clarify its relative strengths, practical advantages, and complementary roles within the broader field of quantitative trait genetics. The utility of this strategy is further demonstrated through comparison with conventional gene prioritization methods.

## 2. Gene Prioritization

### 2.1. Conventional Methods

Table 1 provides an overview of conventional gene prioritization methods that have been developed for chickens and evaluates their advantages and limitations. These approaches can be broadly categorized into six approaches: position-based prioritization, expression-based prioritization, functional annotation, coding variant prediction, non-coding variant prediction, and integrative analysis. Each approach is briefly outlined below.

Position-based prioritization relies primarily on the physical proximity of genes to QTL or GWAS peaks and is the most direct strategy for identifying candidate genes. A representative example in chickens is the study of Zhu et al. [14], which refined chicken QTL regions using a 16th-generation advanced intercross line (AIL) originating from an initial F_2_ population produced by crossing two growth-divergent lines. This AIL was maintained under random mating for 15 years with an average of 1292 individuals per generation, making its maintenance highly labor-intensive and costly. The AIL concept, originally proposed by Davasi and Soller [23], increases recombination events across successive generations, thereby improving QTL mapping resolution and enabling finer localization of QTLs than conventional QTL analysis. Zhu et al. [14] identified tissue-specific regulatory variants and gene networks, revealing conserved functions and distinct regulatory mechanisms compared to mammals. Although position-based prioritization is simple and effective for narrowing down genomic regions, it may fail to capture causal genes located outside the defined regions, i.e., genes regulated through long-range chromatin interactions.

Expression-based prioritization identifies candidate genes through the co-localization of QTLs and loci affecting gene expression. Weighted Gene Co-expression Network Analysis (WGCNA) is a widely used systems biology tool for this purpose. WGCNA identifies groups of genes (modules) exhibiting similar expression patterns by constructing weighted correlation networks and associates these modules with external traits (phenotypes). This approach helps elucidate meaningful gene networks and potential regulatory hub genes. Fan et al. [15] integrated GWAS results with WGCNA in chicken breast muscle, revealing key regulatory genes and networks associated with fatty acid composition. Using genotyping-by-sequencing data from 721 chickens and transcriptome profiles at 14, 22, and 30 weeks of age, they identified nine hub genes, enolase 1 (alpha) (*ENO1*), alcohol dehydrogenase 1C (class I), gamma polypeptide (*ADH1*), N-acylsphingosine amidohydrolase (acid ceramidase) 1 (*ASAH1*), alcohol dehydrogenase 1C (class I), gamma polypeptide (*ADH1C*), phosphatidylinositol-4,5-bisphosphate 3-kinase catalytic subunit delta (*PIK3CD*), WNT1 inducible signaling pathway protein 1 (*WISP1*), AKT serine/threonine kinase 1 (*AKT1*), pantothenate kinase 3 (*PANK3*), and C1q and TNF related 2 (*C1QTNF2*). Similarly, the Chicken Genotype-Tissue Expression (ChickenGTEx) project provides a comprehensive resource of tissue-specific gene expression and eQTLs across 28 chicken tissues, enabling the identification of regulatory variants affecting quantitative traits [16]. The ChickenGTEx portal integrates molecular QTLs associated with transcriptomic phenotypes (RNA-seq analysis, regulatory elements, and context- or environment-dependent regulatory heterogeneity), facilitating the exploration of genetic regulation across multiple tissues [7]. These studies demonstrate how expression-based approaches bridge the gap between QTLs and biological function. However, such analyses require extensive transcriptomic and genotypic datasets from the same individuals, making this approach resource-intensive.

Functional annotation prioritizes candidate genes based on gene function primarily associated with specific phenotypes. This is typically performed by combining differentially expressed genes from transcriptome analysis with functional enrichment tools such as Gene Ontology (GO) and Kyoto Encyclopedia of Genes and Genomes (KEGG). For example, Fu et al. [17] performed a GWAS using whole-genome sequencing data to identify candidate genes associated with egg production traits and elucidated the relevant biological pathways related to these genes through KEGG and GO analyses. Kianpoor et al. [19] integrated GWAS with Gene Set Enrichment Analysis to investigate cell-mediated immunity in chickens, revealing key immune-related genes and pathways. Furthermore, Pan et al. [18] integrated epigenome and transcriptome data across 47 tissues and performed functional annotation using GO and KEGG. This provided a comprehensive functional annotation of the chicken genome, leading to the construction of a tissue-specific regulatory atlas. This atlas describes where genes and regulatory elements are activated within the genome, contrasting with the ChickenGTEx project, which explains how genetic variation affects gene expression patterns. Although functional annotation is a powerful approach, poorly annotated or non-coding genes may still be overlooked.

Coding variant prediction focuses on identifying potentially functionally deleterious amino acid substitutions. For example, Derks et al. [20] annotated missense variants in commercial layer populations using SIFT and PROVEAN, identifying potentially deleterious coding variants that may influence production and health traits. However, the accuracy of such predictions depends on genome annotation quality and evolutionary conservation, and they may overlook context-dependent functional effects such as tissue specificity or developmental timing. Experimental validation is often required to determine the true biological impact of predicted deleterious mutations.

Non-coding variant prediction prioritizes regulatory variants affecting gene expression rather than protein structure. In chickens, this approach is primarily implemented using chicken Combined Annotation–Dependent Depletion (chCADD), which evaluates the functional importance of variants in conserved non-coding regions by integrating multiple genomic annotations, including evolutionary constraints, chromatin features, and predicted regulatory element activity [21]. chCADD assigns a quantitative score that reflects the likelihood that a given variant is deleterious or functionally important, thereby enabling genome-wide prioritization of potentially causal regulatory variants. Additional support comes from eQTL analyses across multiple tissues, including those generated by the ChickenGTEx project [7,16,18]. Methods such as DNase-seq or ChIP-seq can provide additional information about chromatin accessibility and transcription factor binding, but their use in large-scale non-coding variant prioritization remains limited in chickens. Non-coding variant interpretation depends heavily on the availability and quality of tissue-specific regulatory datasets.

Integrative analysis combines multiple omics data to identify candidate genes and elucidate regulatory mechanisms. Shen et al. [22] integrated QTL mapping, 3D genomics, epigenomics, and transcriptomics to identify genes regulating abdominal fat in chickens. They identified two genes, insulin-like growth factor binding protein 2 (*IGFBP2*) and insulin-like growth factor binding protein 5 (*IGFBP5*), as key regulators, demonstrating that specific variants affect transcription factor binding and gene expression, ultimately influencing fat deposition. This example illustrates how multi-omics integration can link genetic variation to complex traits. However, such approaches are limited by high cost, complex data integration, and the need for large sample sizes to obtain robust results, potentially limiting their use to well-resourced research environments.

In addition to these multi-omics strategies, Transcriptome-Wide Association Studies (TWAS) have become a widely adopted framework for integrating GWAS data with gene-expression prediction models trained in an independent reference panel, such as GTEx [9]. In a typical TWAS workflow, eQTL analysis in the reference panel is first used to identify SNPs regulating gene expression, deriving SNP-based prediction weights for each gene. These weights are then applied to genotype data from the GWAS cohort to impute genetically regulated expression. Finally, the association between predicted gene expression and the phenotype is tested. A significant association suggests that genetically driven expression variation in the gene may contribute to the trait. Several extensions and modified TWAS frameworks have been proposed in humans, as reviewed by Shao et al. [24].

In chickens, the application of TWAS remains limited but is progressing. Using a TWAS approach that integrates GWAS results with the ChickenGTEx reference, Zhong et al. [25] reported genetic variants associated with body weight across three growth stages from hatching to 72 weeks of age in an F_2_ chicken population derived from a cross between the WL and the Dongxiang breeds. However, the use of TWAS in chickens and livestock remains constrained by the lack of large-scale eQTL reference panels comparable to those available in humans. Furthermore, because TWAS relies on statistical prediction rather than direct gene expression measurements in GWAS cohorts, false positives may arise due to LD structures or tissue mismatches between the reference panel and the target population.

Compared with TWAS, the integrated F_2_-based strategy proposed in the present study uses genotypes, expression levels, and phenotypic values measured directly from the same individuals. This enables verification of causality without the need for large-scale external eQTL reference datasets, making it applicable to species with limited genomic resources and populations with unique genetic backgrounds not represented in existing reference panels.

The six approaches outlined above are not mutually exclusive; rather, they can be applied in a complementary and integrated manner. In humans, four gene prioritization methods have been established: gene-based association tests, integrative analysis of GWAS and molecular QTL data, enhancer-gene connection maps, and network-based gene prioritization. Their methodologies are comprehensively reviewed by Qi et al. [8].

### 2.2. New Integrated Genetic Method

Based on principles of population genetics and quantitative genetics, I developed a new, hypothesis-free, and systematic approach in chickens. This framework consists of six sequential steps: QTL remapping, RNA-seq analysis, RT-qPCR validation, haplotype frequency comparison, association analysis, and conditional correlation analysis [12]. A major advantage of this strategy is that it does not require the establishment of additional crossbred chicken lines, such as congenic lines or the aforementioned AIL, for fine mapping of QTL regions following initial QTL detection. Instead, the method utilizes the same F_2_ segregating population used in the original QTL analysis. By sampling tissues or organs most relevant to the QTL phenotypes for transcriptome analysis, this approach enables the efficient and cost-effective identification of the most plausible candidate genes.

Table 2 outlines the proposed integrated genetic approach and summarizes the materials and objectives used in each of the six sequential steps. Briefly, Step 1 involves remapping a QTL identified in the initial QTL analysis using traits that show significant QTL effects to refine the 95% confidence interval (CI) as precisely as possible. In many cases, multiple traits are affected by a single QTL. In the chicken study by Ochiai et al. [12], seven traits measured in an open field test were affected by a single QTL on chromosome 4. Principal component analysis (PCA) was therefore applied to summarize the shared variances among these seven traits. The first principal component (PC1) explained 90.2% of the total trait variance, and PC1 scores were subsequently used as a composite trait for QTL remapping. When multiple traits are significantly affected by the same QTL, the use of such a composite trait enables the accurate ranking and selection of F_2_ individuals exhibiting two distinct extreme phenotypes for subsequent analyses.

In Step 2, RNA-seq analyses are performed using individuals with extreme phenotypic values from the F_2_ population employed in the initial QTL analysis. Ochiai et al. [12] used pooled RNA samples from the three highest- and three lowest-ranking F_2_ individuals to reduce experimental costs. This screening is expected to identify dozens of differentially expressed genes (DEGs) located within the 95% CI of the QTL, which typically spans several tens of megabases and encompasses hundreds of genes. To minimize false-negative exclusion of potentially causal genes, relatively low fold-change and statistical significance thresholds are intentionally applied. Because most QTLs explain less than 10% of the phenotypic variance, causal genes are unlikely to exhibit large gene expression differences, such as changes exceeding two-fold [5,8,13]. Consequently, applying a stringent fold-change cutoff at the initial RNA-seq step carries a substantial risk of prematurely excluding true causal candidates. Although permissive thresholds inevitably increase the number of false-positive DEGs at this stage, this limitation is explicitly addressed through multiple downstream validation steps incorporated into the integrative framework, as discussed below.

In the study by Ochiai et al. [12], RNA-seq analysis using low fold-change thresholds (>1.2-fold and <0.83-fold) identified 35 DEGs among 333 genes located within the 95% CI of the QTL. These candidate genes were subsequently subjected to independent validation and filtering in later analytical steps, thereby substantially reducing the likelihood that false-positive genes were retained. With the recent reduction in RNA-seq costs, individual-level RNA-seq analysis is now recommended whenever feasible, as it provides greater statistical power and flexibility than pooled RNA approaches.

In Step 3, RT-qPCR analyses are performed using at least 10 individuals from each of the two parental breeds or lines, as well as their F_1_ progeny, to validate the expression patterns of DEGs detected by RNA-seq. To ensure objectivity and independence, these individuals should be distinct from those used for constructing the F_2_ population for QTL mapping. In Ochiai et al. [12], 16 of the 35 DEGs detected by RNA-seq were successfully validated by RT-qPCR at a nominal significance level of *p* < 0.05, as determined by one-way analysis of variance (ANOVA) followed by Tukey’s honestly significant difference test.

In Step 4, haplotype frequencies at DEG loci that passed the RT-qPCR validation are compared between two extreme F_2_ groups (approximately 20 individuals each with the highest and lowest phenotypic values). Individuals carrying recombined haplotypes are excluded from this analysis. If causal genes exist among the DEGs, their haplotype frequencies are expected to differ significantly between the two extreme groups, allowing exclusion of genes unrelated to the phenotype. Using 20 high- and 19 low-ranking individuals from the F_2_ population derived from the NAG and WL-G breeds, Ochiai et al. [12] successfully narrowed the number of candidate genes from 16 to 11 (nominal *p* < 0.05, Pearson’s chi-square test).

In Step 5, expression levels of the DEGs that passed haplotype frequency analysis are compared between two extreme F_2_ groups (approximately 20 individuals each). From the 11 DEGs that passed RT-qPCR validation, Ochiai et al. [12] identified two genes, an uncharacterized gene (*LOC101749214*) and neuropeptide Y receptor Y5 (*NPY5R*). *LOC101749214* showed a significant difference in expression between the two groups (*p* < 0.05, Student’s *t*-test), whereas *NPY5R* exhibited a marginal difference (*p* = 0.060).

The sample sizes of the extreme groups used in Steps 4 and 5 were determined based on the selective DNA pooling method proposed by Darvasi and Soller [26]. This approach combines selective genotyping with DNA pooling as a cost-effective QTL mapping strategy by sampling individuals from the phenotypic extremes. According to their theoretical analysis, selecting approximately 10% of individuals from each extreme phenotype group provides an appropriate balance between statistical power and experimental efficiency.

As the final Step 6, to control for diplotype effects on the phenotype, conditional correlation analysis is performed between phenotypic values and expression levels of the DEGs that passed the association analysis. In the study by Ochiai et al. [12], to evaluate the discriminatory power of this analysis alone, all 11 DEGs that passed the RT-qPCR validation were subjected to conditional correlation analysis. Among these DEGs, only *NPY5R* expression exhibited a significant positive correlation with open-field activity (*p* = 0.023), whereas *LOC101749214* expression showed a marginal negative correlation (*p* = 0.059). *LOC101749214* is functionally uncharacterized, whereas *NPY5R* is well known for its role in hypothalamic regulation of feeding behavior [27] and has recently been implicated in emotional modulation [28]. Consequently, Ochiai et al. [12] identified two strong candidate genes, *LOC101749214* and *NPY5R*, from 333 genes within a 21-Mb interval on chicken chromosome 4. These findings suggest that comparing expression in Step 5 may not always be essential, whereas conditional correlation analysis serves as a critical causal inference test.

The proposed integrative framework (Table 2) incorporates multiple opportunities for cross-validation to reduce the risk of bias accumulation inherent in stepwise inference. First, RT-qPCR analysis in Step 3 constitutes cross-population validation because it uses parental and F_1_ populations rather than the F_2_ population used in RNA-seq analysis. Second, haplotype frequency analysis in Step 4 provides cross-experimental validation, as haplotypes are evaluated as a genetic trait independent of gene expression. Third, association analysis in Step 5 increases the effective sample size relative to the RNA-seq, thereby strengthening validation of DEGs identified in Step 2.

With respect to significance thresholds, relatively permissive nominal *p*-value cutoffs were intentionally adopted throughout the analytical pipeline to minimize false-negative exclusion of potentially causal genes during early screening. In Step 2, multiple testing-adjusted *p* values could not be applied because pooled RNA samples were used for RNA-seq analysis in the study by Ochiai et al. [12]. In subsequent steps, nominal significance levels (*p* < 0.05) and marginal thresholds were used instead of overly conservative corrections such as the Bonferroni adjustment. Excessively stringent thresholds can markedly reduce the number of retained candidate genes and may eliminate true causal genes at early stages of analysis. This effect is illustrated by reanalyzing the dataset of Ochiai et al. [12] using Bonferroni-corrected thresholds. In Step 3, the number of genes significant at nominal *p* < 0.05 decreased from 16 to eight at a Bonferroni-corrected threshold of *p* < 0.00147 (=0.05/34), excluding *LOC101749214* while retaining *NYP5R*. In Step 4, 11 genes significant at nominal *p* < 0.05 were reduced to zero at a Bonferroni-corrected threshold of *p* < 0.00313 (=0.05/16). These results demonstrate that excessively stringent thresholds may prematurely eliminate true causal genes. Collectively, these design features ensure that candidate genes are not selected based on a single analytical criterion but are instead supported by consistent evidence across multiple, partially independent analyses. This balanced strategy minimizes false negatives while maintaining robustness, and final causal inference can be further strengthened through downstream causal analysis and quantitative complementation testing.

The proposed strategy can be readily applicable to segregating F_2_ populations derived from crosses between diverse chicken lines, including commercially selected lines and local indigenous breeds, as well as to populations obtained from crosses involving other small livestock breeds and model organism strains. For example, when two chicken lines are divergently selected from a common base population for opposite phenotypic traits, they tend to carry different alleles at QTLs located in line-specific chromosomal regions, while sharing the same alleles at QTLs on chromosomal regions common to both lines. Consequently, in the F_2_ population generated from a cross between these two lines, only a subset of QTLs segregates, thereby simplifying the genetic architecture of the trait variation. This reduction in genetic complexity increases the statistical power of the proposed strategy for detecting candidate genes.

Another advantage is that the strategy reuses the same F_2_ population employed in the original QTL mapping. Even if the sample size of this population is limited, the target QTL has already been successfully identified at the genome-wide 5% significance level using that sample size. Therefore, the likelihood that the present integrative strategy fails due to insufficient sample size is considered low. However, when the target QTLs are suggestive loci that do not exceed the genome-wide 5% significance threshold, the strategy may not work effectively.

In addition, when the parental breeds used to develop the F_2_ population are unavailable, the RT-qPCR validation step (Step 3) may be omitted. In such cases, validation of DEGs can be conducted in Steps 4 to 6 (Table 2), followed by causal analysis (see the next section).

In summary, the study by Ochiai et al. [12] demonstrates that the proposed integrated genetic approach can efficiently narrow down candidate genes to a very small number in a cost- and time-effective manner, without requiring additional fine-mapping of the broad initial QTL interval through further crossbreeding. A major strength of this approach lies in its systematic integration of multiple analytical steps applied to a single segregating population. Because phenotypic measurements, molecular data, and genotypes are all obtained from the same F_2_ population, relationships between genotypes, molecular traits, and phenotypes can be evaluated in a unified and internally consistent framework. Moreover, the framework incorporates multiple opportunities for cross-validation and adopts permissive early-stage thresholds to reduce false-negative exclusion of causal genes. Finally, this hypothesis-independent strategy enables identification of candidate genes with both known and unknown functions, thereby overcoming the limitations imposed by incomplete functional annotation in conventional gene prioritization methods (Table 1).

Another example of applying this integrated strategy is a study on chickens concerning a QTL on chromosome 2 associated with breast muscle weight at four weeks of age, using 239 F_2_ chickens derived from a cross between NAG and White Plymouth Rock breeds by Furuta and Ishikawa [29]. In that study, QTL remapping followed by RNA-seq analysis identified 23 DEGs among 329 genes located within the 95% CI of the QTL. Gene enrichment analysis suggested GATA binding protein 6 (*GATA6*) as a functional candidate; however, subsequent RT-qPCR analysis excluded *GATA6* from the candidate list. Following haplotype frequency and correlation analyses, cadherin-17 (*CDH17*) was ultimately identified as the primary candidate gene, with ring finger protein 151 (*RNF151*) identified as a secondary candidate gene.

To date, the proposed strategy has been successfully applied in only two studies: Ochiai et al. [12] and Furuta and Ishikawa [29]. However, because the traits examined in those studies—behavior [12] and muscle weight [29]—have markedly different biological backgrounds, this strategy is fundamentally applicable to any quantitative trait, including those that follow continuous or binary distributions.

## 3. Causal Gene Identification

### 3.1. Causal Analysis

Causal analysis evaluates whether the expression of a candidate gene causally mediates the relationship between genotype and phenotype. At the genome-wide analysis level, Mendelian randomization using genetic variants as instrumental variables is commonly employed to assess causal relationships between molecular traits (as exposures) and phenotypic outcomes [30,31,32]. However, our integrative genetic approach allows us to narrow the list of candidate genes down to only a few genes. This enables manual application of the Causal Inference Test (CIT), a causal analysis method based on a simple statistical framework [33], without requiring the development of specialized software.

**Figure 1:**
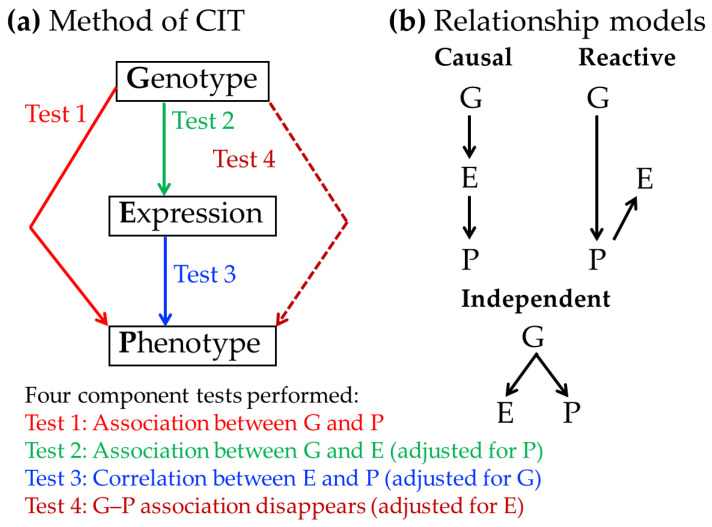
Method of the Causal Inference Test (CIT) and relationship models. (**a**) The CIT framework evaluates causal relationships among genotype (G), gene expression (E), and phenotype (P) using four component tests (see text for details); (**b**) Three possible relationship models among G, E, and P: Causal (G → E → P), Reactive (G → P → E), and Independent (G independently influences E and P).

As illustrated in Figure 1a, the CIT evaluates a causal model in which the effect of genotype (G) on phenotype (P) is mediated through gene expression (E). The framework comprises four statistical tests:

Test 1 examines whether G is significantly associated with P.

Test 2 evaluates whether G remains significantly associated with E after adjusting for P.

Test 3 determines whether E is significantly correlated with P after adjusting for G.

Test 4 assesses whether the association between G and P disappears after adjusting for E.

Based on the outcomes of these tests, the CIT distinguishes three possible relationship modes among G, E, and P (Figure 1b): causal, reactive, and independent. In the causal model, G influences P through E. In the reactive model, E changes in response to variation in P. In the independent model, G affects both E and P independently. When E satisfies the criteria for the causal model, the corresponding gene is inferred to be the causal gene mediating the relationship between G and P.

There are three major advantages of using the CIT. First, its four component tests enable a clear distinction between genes showing true causal relationships and those exhibiting reactive or independent relationships, thereby filtering out consequential genes. Second, the CIT requires no a priori assumptions during testing because each component test is based on conditional correlation analysis. Third, the CIT can reveal potential pleiotropic effects of a gene on the phenotype by identifying independent pathways linking genotype, expression, and phenotype. Therefore, the CIT can substantially reduce the number of candidate QTL genes and may facilitate the discovery of genes, including those with previously unrecognized functions that affect the phenotype.

The CIT has several limitations that should be considered when interpreting its results, as reviewed by Ishikawa [5]. First, population stratification can confound causal inference. However, this is unlikely to be a major concern in chickens and other model animals, such as mice, in which well-controlled segregating populations (e.g., F_2_ populations) are typically constructed for CIT analyses [5,13].

Second, spurious causal mediation may arise when the genetic locus responsible for the tested mediator is tightly linked to that of an unmeasured true causal mediator (i.e., both loci for mediators are in linkage disequilibrium), potentially leading to incorrect inference. To mitigate this issue, the conditional correlation analysis employed in Step 6 of Table 2 (corresponding to Test 3 in Figure 1) explicitly evaluates the relationship between gene expression and phenotype while controlling for diplotype effects. This helps distinguish associations attributable to direct mediation from those caused by linked loci.

Third, the statistical power of CIT depends on the strength of the association between genotype and gene expression. When genotype–expression associations are weak, as is often the case for regulatory variants underlying QTLs with small phenotypic effects, CIT may only marginally detect true causal mediation or fail to distinguish among multiple potential mediations.

Given these limitations, CIT should not be used as a standalone criterion but rather as Step 7 within the present integrative framework, where prior filtering steps enrich for genes with biologically meaningful genotype–expression relationships. These limitations can be effectively addressed by quantitative complementation testing, as described in the next section.

### 3.2. Quantitative Complementation Test

**Figure 2:**
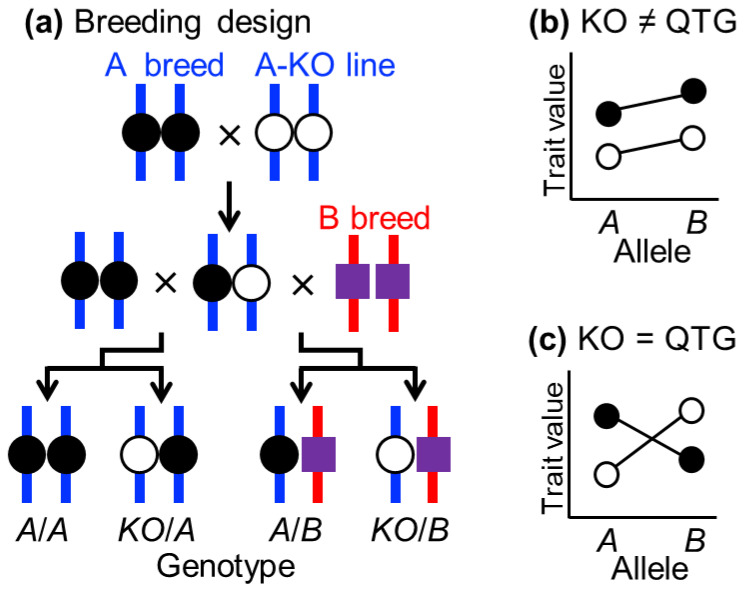
Schematic diagram of the Quantitative Complementation Test (QCT) using knockout (KO) chickens. (**a**) Breeding design for developing two segregating populations using three breeds: the A breed is homozygous for the normal *A* allele (closed circle) at the QTL, the knockout line A-KO carries the *KO* allele (open circle), and the B breed carries the normal *B* allele (purple square) at the QTL; the A and A-KO breeds share the same chromosomal background (blue vertical bars) except for the *KO* locus, whereas the B breed has a different chromosomal background (red vertical bars); (**b**) When the KO locus differs from the QTL, no significant interaction is observed between the QTL and the *KO* locus for the trait across the two genetic backgrounds (homozygous for A chromosomes and heterozygous for A and B chromosomes); (**c**) When the *KO* locus and the QTL are identical, a significant interaction between the QTL and the *KO* locus is observed; QTG, quantitative trait gene.

The strongest evidence that a candidate gene is the actual causal gene for a target QTL is obtained by performing a Quantitative Complementation Test (QCT) using knockout animals for the candidate gene [13,34,35]. Figure 2 illustrates the schematic procedure of QCT. Starting from one of the parental chicken breeds used for QTL analysis (the A breed in this example), a new knockout line (A-KO) is generated by disrupting the candidate gene on the same genetic background as the A breed using CRISPR/Cas9 or other genome editing tools (Figure 2a). Crossing the A and A-KO breeds produces F_1_ progeny heterozygous for the normal *A* and knockout (*KO*) alleles. These F_1_ birds are then backcrossed to both A and B breeds, producing two segregating backcross populations. One population segregates for the *A* and *KO* alleles on the pure A-breed genetic background (blue vertical bars). The other population segregates for these alleles on a genetic background heterozygous for the A and B breeds (blue and red vertical bars). Importantly, except for the candidate gene locus, all alleles at all QTLs on other chromosomal regions become fixed in either a homozygous or heterozygous state.

When the *KO* locus differs from the target QTL, a two-way analysis of variance (ANOVA) fails to detect a significant interaction effect between the QTL and the *KO* locus on trait values (Figure 2b). In contrast, when the *KO* locus corresponds to the QTL, two-way ANOVA reveals a significant interaction effect between the QTL and the *KO* locus (Figure 2c), thereby indicating that the *KO* gene is the true causal quantitative trait gene (QTG).

It is common to identify multiple candidate genes within the 95% CI of a QTL, and these gene loci are typically tightly linked to each other (i.e., in LD). Chen et al. [35] performed QCT on six QTLs associated with fear-related behaviors in mice and obtained highly informative results. Within one QTL region on mouse chromosome 13, they identified five closely located genes as candidates. Among these, hyperpolarization-activated cyclic nucleotide-gated potassium channel 1 (*Hcn1*) had previously been proposed as a functional causal gene based on evidence that pharmacological blockade of HCN1 reduces freezing behavior. However, QCT using *Hcn1* knockout mice excluded *Hcn1* from causality and instead identified the unannotated gene *4933413L06Rik* as the causal gene. This clearly demonstrates the ability of QCT to pinpoint the causal gene locus underlying a QTL from tightly linked neighboring loci. Similarly, in our previous mouse study [13], following CIT, QCT successfully identified lymphocyte antigen 75 (*Ly75*) as the causal gene for a QTL affecting white fat weight on mouse chromosome 2.

Together, QCT provides definitive genetic evidence for causality. The next step is to identify causal genetic variants within the causal genes revealed by QCT and, through multi-omics approaches such as genomics, transcriptomics, proteomics, and metabolomics, to fully elucidate the genetic basis of phenotypic traits at multiple biological levels. The resulting molecular insights into complex quantitative traits will not only facilitate precise genetic improvement in various livestock species, including chickens, but also hold promise for broader applications to human traits and diseases.

## 4. Conclusions

The integrated genetic approach developed in chickens combines QTL remapping, transcriptome analysis, haplotype frequency comparison, association analysis, and conditional correlation analysis into a unified analytical framework. By applying this series of analyses to the original F_2_ segregating population used for QTL mapping, the approach enables efficient and hypothesis-independent identification of a small number of candidate genes without requiring additional fine-mapping of QTL regions through further crossbreeding. This framework thus provides a powerful and cost-effective strategy for gene prioritization in chickens.

The utility of this approach is further enhanced by integrating it with causal analysis in the F_2_ population and quantitative complementation tests using knockout birds, which together enable definitive identification of causal genes underlying quantitative traits. The strategy is readily applicable to any segregating F_2_ population obtained from crosses between diverse chicken lines, including commercially selected lines and local indigenous breeds. Furthermore, this conceptual framework can be extended to other small livestock species and model organisms where the construction and utilization of segregating populations is feasible.

## Figures and Tables

**Table 1:** Systematic evaluation of conventional gene prioritization methods in chickens following GWAS and QTL analyses.

Method	Main Approach	Advantage	Limitation	Reference
Position-based prioritization	Fine mapping of QTL/GWAS regions; identification of nearest genes or those within LD blocks	Simple and straightforward; directly links genomic regions to genes	Difficult to narrow down to a single causal gene; labor- and cost-intensive	[14]
Expression-based prioritization	eQTL analysis; differentially expressed genes (DEGs); co-expression (e.g., WGCNA ^1^)	Links gene expression to traits; provides tissue-specific insights	Requires RNA from the same population; sensitive to tissue environment; resource- and cost-intensive	[15,16]
Functional annotation	GO/KEGG ^2^ enrichment; tissue-specific expression	Provides biological context and functional clues	May overlook poorly annotated or non-coding genes; broad or indirect terms	[17,18,19]
Coding variant prediction	In silico prediction of functional impact of nonsynonymous variants using tools such as SIFT ^3^ or PROVEAN ^4^	Identifies potentially damaging coding variants within candidate genes	Limited to coding regions; may miss non-coding effects	[20]
Non-coding variant prediction	Annotation of regulatory regions using chCADD ^5^ or eQTL	Prioritizes non-coding regulatory variants affecting gene expression	Dependent on available datasets; regulatory mechanisms may differ by tissue	[7,18,21]
Integrative analysis	Combines QTL mapping, transcriptomics (e.g., TWAS ^6^)	Enables identification of candidate genes and regulatory mechanisms	Computationally intensive; requires large and well-matched datasets	[22]

^1^ WGCNA constructs weighted gene co-expression networks to detect biologically relevant gene modules. ^2^ GO/KEGG pathway enrichment identifies statistically overrepresented biological processes and pathways in gene sets. ^3^ SIFT predicts whether amino acid substitutions caused by missense variants are deleterious. ^4^ PROVEAN scores the impact of missense variants to classify them as deleterious or tolerated. ^5^ chCADD scores variants in conserved non-coding elements in the genome. ^6^ TWAS tests trait associations between phenotypes and genetically predicted gene expression derived from eQTL reference panels.

**Table 2:** Overview of new integrative genetic approach in chickens.

Step	Methods	Materials	Objective
1	QTL remapping	SNP markers and phenotypic data from the segregating F_2_ mapping population	Refine the QTL 95% confidence interval (CI) with higher precision
2	RNA-seq analysis	RNA from three F_2_ individuals with extreme (top and bottom) phenotypes	Identify differentially expressed genes (DEGs) within the CI
3	RT-qPCR validation	RNA from parental breeds and F_1_ individuals (*n* = 10 each)	Validate DEG expression patterns in populations different from the population used in RNA-seq analysis
4	Haplotype frequency analysis	Haplotypes from two extreme F_2_ groups (*n* = 20 each)	Compare haplotype frequencies of validated DEGs between groups; use haplotype frequencies as a trait distinct from gene expression for validation
5	Association analysis	Gene expression data from the two extreme groups	Test expression differences between groups; validate DEG expression patterns in RNA-seq analysis
6	Conditional correlation analysis	Gene expression, diplotypes, and phenotypes from the two extreme groups	Assess expression–phenotype correlation conditioned on diplotypes

## Data Availability

No new data were created or analyzed in this study.

## Abbreviations

The following abbreviations are used in this manuscript:

QTL	quantitative trait locus
GWAS	genome-wide association study
LD	linkage disequilibrium
eQTL	expression QTL
RT-qPCR	reverse transcription quantitative polymerase chain reaction
NAG	Nagoya
WL	White Leghorn
DEG	differentially expressed gene
AIL	advanced intercross line
WGCNA	weighted gene co-expression network analysis
*ENO1*	enolase 1, (alpha)
*ADH1*	alcohol dehydrogenase 1C (class I), gamma polypeptide
*ASAH1*	N-acylsphingosine amidohydrolase (acid ceramidase) 1
*ADH1C*	alcohol dehydrogenase 1C (class I), gamma polypeptide
*PIK3CD*	phosphatidylinositol-4,5-bisphosphate 3-kinase catalytic subunit delta
*WISP1*	WNT1 inducible signaling pathway protein 1
*AKT1*	AKT serine/threonine kinase 1
*PANK3*	pantothenate kinase 3
*C1QTNF2*	C1q and TNF-related 2
ChickenGTEx	Chicken Genotype-Tissue Expression
GO	Gene Ontology
KEGG	Kyoto Encyclopedia of Genes and Genomes
chCADD	chicken Combined Annotation–Dependent Depletion
*IGFBP2*	insulin-like growth factor binding protein 2
*IGFBP5*	insulin-like growth factor binding protein 5
TWAS	Transcriptome-Wide Association Studies
SNP	Single-nucleotide polymorphism
CI	confidence interval
PCA	principal component analysis
PC1	the first principal component
ANOVA	analysis of variance
*NPY5R*	neuropeptide Y receptor Y5
CIT	Causal Inference Test
QCT	Quantitative Complementation Test
KO	knockout
QTG	quantitative trait gene
*Hcn1*	hyperpolarization-activated cyclic nucleotide-gated potassium channel 1
*Ly75*	lymphocyte antigen 75
